# Communication Inequalities, Social Determinants, and Intermittent Smoking in the 2003 Health Information National Trends Survey

**Published:** 2009-03-15

**Authors:** Leland K. Ackerson, Kasisomayajula Viswanath

**Affiliations:** Department of Community Health and Sustainability, University of Massachusetts Lowell. At the time of this research, Dr Ackerson was affiliated with the Dana-Farber Cancer Institute, Boston, Massachusetts; Harvard School of Public Health and the Dana-Farber Cancer Institute, Boston, Massachusetts

## Abstract

**Introduction:**

Intermittent smokers account for a large proportion of all smokers, and this trend is increasing. Social and communication inequalities may account for disparities in intermittent smoking status.

**Methods:**

Data for this study came from 2,641 ever-smokers from a 2003 nationally representative cross-sectional survey. Independent variables of interest included race/ethnicity, sex, household income, education, health media attention, and cancer-related beliefs. The outcome of interest was smoking status categorized as daily smoker, intermittent smoker, or former smoker. Analyses used 2 sets of multivariable logistic regressions to investigate the associations of covariates with intermittent smokers compared with former smokers and with daily smokers.

**Results:**

People with high education and high income, Spanish-speaking Hispanics, and women were the most likely to be intermittent rather than daily smokers. Women and Spanish-speaking Hispanics were the most likely to be intermittent rather than former smokers. Attention to health media sources increased the likelihood that a person would be an intermittent smoker instead of a former or daily smoker. Believing that damage from smoking is avoidable and irreversible was associated with lower odds of being an intermittent smoker rather than a former smoker but did not differentiate intermittent smoking from daily smoking.

**Conclusion:**

The results indicate that tailoring smoking-cessation campaigns toward intermittent smokers from specific demographic groups by using health media may improve the effect of these campaigns and reduce social health disparities.

## Introduction

The proportion of smokers who are nondaily smokers is rising, climbing from 9.3% in 1994 to 23.7% in 2006 (1). Although low-level smokers are less likely than heavy smokers to assess their smoking as risky and are less committed to cessation (2), long-term nondaily or intermittent smoking increases cancer risk (3). In addition, intermittent smoking often leads to daily smoking (4,5).

Intermittent smokers tend to be female, Hispanic, highly educated, and unmarried (5-7). However, with a notable exception (7), little research has used nationally representative samples of adult smokers. Previous research has also tended to study the association between intermittent smoking status and a single socioeconomic variable rather than investigating multiple measures.

Despite increasing emphasis on the role of social disparities in health (8), much work remains to be done in mapping the pathways between social characteristics and health outcomes. The structural influence model (SIM) suggests health communication factors can be a pathway that may link social determinants such as race/ethnicity and class with health outcomes (9). SIM emphasizes that media communications influence health by raising awareness, focusing attention, framing issues, providing information, and reinforcing knowledge, attitudes, and behaviors. SIM acknowledges that different forms of mass media and different genres within a medium may differentially influence behaviors. SIM indicates that social disparities and social patterning in behavioral norms in different communities deter access to some information sources and encourage access to others. SIM proposes that communication inequalities may affect additional dimensions such as attention to, processing of, and acting on health information (10). That media may affect health behaviors is evidenced by earlier research linking media use to smoking (11).

We used a nationally representative sample of smokers in an exploratory analysis to 1) shed light on how social inequalities characterize intermittent smokers and 2) test the portion of SIM that proposes links between communication inequalities and health outcomes.

## Methods

The data for this study came from the 2003 Health Information National Trends Survey, a nationally representative cross-sectional study of health media use and cancer-related knowledge among adults in the United States (12). After random-digit–dialing selection (response rates of 55% for a household screener and 63% for the interview), 6,396 participants completed a telephone interview. We restricted the analysis to those who reported that they had smoked at least 100 cigarettes, termed ever smokers, leaving 2,927 participants. We removed participants who were missing information regarding covariates, for a final sample of 2,641.

The outcome of interest was a 3-category smoking measure. Trained interviewers asked participants who reported smoking at least 100 cigarettes in their entire life, "Do you now smoke cigarettes every day, some days, or not at all?" We considered respondents who endorsed these options to be daily smokers, intermittent smokers, and former smokers, respectively. In this study, we were interested in investigating adult influences on smoking behavior. In the United States, smoking initiation occurs predominantly among minors (13), so we considered never-smokers in this age range to be in a categorically different risk group. Therefore, we excluded those who had not smoked 100 cigarettes from our analyses.

We considered socioeconomic and demographic variables linked to health disparities in smoking (5,7) and media use (9,10), including education, annual household income, race/ethnicity, and sex. We categorized education as less than high school graduate, high school graduate, some college, or college graduate. We categorized annual household income as less than $25,000, $25,000 to $34,999, $35,000 to $49,999, $50,000 to $74,999, or $75,000 and above. We created the race/ethnicity measure after preliminary analyses indicated differences in smoking patterns between Hispanics interviewed in English and Spanish by combining 2 constructs, ethnicity and language of interview. This combination of self-reported categorization and selection of interview language yielded 5 categories: non-Hispanic white, English-speaking Hispanic, Spanish-speaking Hispanic, non-Hispanic black, and non-Hispanic other.

Health communication variables in this analysis included cancer information seeking and attention paid to health information in mass media (14). We measured information seeking with the question, "Have you ever looked for information about cancer from any source?" We assessed attention paid to health media messages separately for each of 5 sources — television, radio, newspapers, magazines, and the Internet — with the question "How much attention do you pay to information about health or medical topics [from each source]? Would you say a lot, some, a little, or not at all?" We collapsed responses to the questions to create 5 binary variables, 1 for each source, to measure whether the person paid a lot of attention ("a lot") or not a lot of attention ("some," "a little," or "not at all") to each source.

We assessed cancer-related beliefs with questions that assessed whether respondents agreed with 6 statements: "Exercise can undo most of the effects of smoking," "Vitamins can undo most of the effects of smoking," "There's no risk of getting cancer if someone only smokes a few years," "Whether a person gets lung cancer depends more on genes than anything else," "It seems like almost everything causes cancer," and "There's not much people can do to lower their chances of getting cancer." We created 6 separate binary predictors, 1 for each statement, by collapsing the responses "disagree" and "strongly disagree" into 1 "disagree" category, hereafter termed "endorsing healthy cancer beliefs," and collapsing the responses "agree," "strongly agree," and "no opinion" into a "does not disagree" category, hereafter termed "endorsing unhealthy cancer beliefs."

We included variables theoretically linked to smoking status as controls in the analyses. These included location of residence (15), marital status (16), age (1), cancer history (17), and family cancer history (17). Consistent with the United States Department of Agriculture Economic Research Service, we categorized location of residence as a county in a metropolitan area with 1 million or more residents; a county in a metropolitan area with fewer than 1 million residents; a county in a nonmetropolitan area with 20,000 or more urban residents; and a county in a nonmetropolitan area with fewer than 20,000 urban residents (18). The binary marital status variable defined respondents as married/cohabiting or not. We categorized age in years in approximately equal-sized groups as 18 to 34; 35 to 49; 50 to 64; 64 to 74; or 75 or more. History of cancer and family history of cancer were binary indicators of whether the respondent or a brother, sister, parent, child, or other close family member had cancer.

We performed all analyses in SUDAAN version 9.0 (RTI International, Research Triangle Park, North Carolina). We assessed unadjusted associations between smoking status and independent variables with χ^2^ tests. We used 2 series of multivariable logistic regressions to examine the adjusted associations between smoking status and all socioeconomic and demographic variables. In 1 series, we compared intermittent smoking with daily smoking as the reference outcome, and in the other series, we compared intermittent smoking with former smoking as the reference outcome. In each model series, we tested each binary health media variable and cancer-related health belief individually as a predictor of smoking status, while keeping all socioeconomic and demographic covariates in the models. All regression analyses used 2-tailed tests with 95% confidence intervals.

## Results

Our sample contained 280 intermittent smokers; this number represented 24.4% of current smokers (Table 1). The sample was approximately evenly distributed among levels of education, although the largest proportion had at least a high school education. Almost half of the sample reported seeking cancer information. Of the unhealthy cancer beliefs, only 1 (almost everything causes cancer) was held by more than half of participants.

In multivariate analysis, college graduates and those from the wealthiest households were more likely to be intermittent smokers than daily smokers, compared with those who did not graduate from high school and those from the poorest households (Table 2). These differences were not seen when comparing intermittent with former smokers. Compared with men, women were more likely to be intermittent than former or daily smokers. Spanish-speaking Hispanics had the highest odds of intermittent smoking, compared with non-Hispanic whites.

Although smoking status was not associated with cancer information seeking (Table 2), it was associated with attention to health media (Table 3). People who paid a lot of attention to health information on the radio were more likely than those who did not pay a lot of attention to be intermittent smokers rather than either daily smokers or former smokers. Compared with those who were not attentive to health information in newspapers, those who paid a lot of attention were more likely to be intermittent rather than former smokers. The results also suggested that those who paid a lot of attention to television and magazines may be more likely to be intermittent rather than daily smokers compared with those who did not pay a lot of attention to these media.

Smoking status was also associated with cancer-related health beliefs (Table 4). Believing that neither exercise nor vitamins can undo the harmful effects of smoking was associated with lower odds of being an intermittent smoker rather than a former smoker. Additionally, believing that lung cancer risk is primarily dependent on genes and believing that nearly everything causes cancer were associated with higher odds of being an intermittent smoker rather than a former smoker. None of the cancer-related health beliefs distinguished intermittent smokers from daily smokers.

## Discussion

Consistent with previous estimates (1), our analyses indicate that nearly one-fourth of current smokers were intermittent smokers in 2003. This finding has implications for smoking cessation programs in the United States that have typically targeted daily smokers (19). Many intermittent smokers do not consider themselves to be smokers, and messages of quitting may not resonate with them (20). Many intermittent smokers go on to become daily smokers (5), but the period of intermittent smoking is an opportunity to intervene before addiction occurs (20,21).

As with previous work (22,23), our analyses found that socioeconomic status differentiates intermittent smokers from daily but not former smokers. Although other studies have found higher education to be associated with intermittent compared with daily smoking (5,24), we included education and income and found that both have independent associations with intermittent smoking. This finding indicates that these constructs may influence smoking through different pathways. Those with higher education may be more aware of the health risks inherent in daily smoking (25), and people with higher incomes may have resources to help them overcome or avoid nicotine addiction (26).

Women are more likely than men to be intermittent smokers rather than daily or former smokers. Women may be more likely to intermittently smoke for extended periods of time, while men may be more likely to begin smoking daily or quit entirely. This hypothesis is consistent with evidence that indicates that women are more likely to be low-level smokers (6) and less likely to increase smoking frequency during college (4). This smoking pattern may reflect gendered smoking norms, such as the use of tobacco by women for weight maintenance (27), or gendered associations with depression (28), as well as the lower success rate for smoking cessation among women (29).

We found that Spanish-speaking Hispanics are more likely than any other ethnic group to be intermittent smokers. This finding is consistent with previous research that found a high prevalence of intermittent smoking among Hispanics of any language preference (5,6). Another study found that more than 70% of Latino smokers in California either did not smoke every day or smoked fewer than 5 cigarettes per day (30). This evidence contradicts traditional withdrawal-based theories of smoking, and we suggest that a culturally based theory that takes into account social smoking norms, beliefs, and expectations of a person's ethnic community may be more appropriate to predict smoking behavior among Hispanics. The finding in our study that English-speaking Hispanics had smoking patterns more similar to those of non-Hispanic whites than to those of Spanish-speaking Hispanics suggests that this theory is less applicable to Hispanics who are comfortable communicating in English and, presumably, are more familiar with and influenced by the culture and traditions of other Americans.

Our findings have several implications regarding health media use and smoking status. Since cancer information seeking did not differentiate intermittent smokers from either daily or former smokers, intermittent smokers probably do not decide to smoke intermittently after researching health effects. If health information influences the decision to smoke, it is most likely due to incidental exposure rather than to active information seeking.

Our findings also indicate that attention to health information from the radio, and perhaps other media, may influence a person to smoke intermittently instead of daily. These results are comparable with those from previous studies that found that lower social participation, interpreted as forms of informational support and social control, is associated with higher levels of daily than intermittent smoking (23,31). Our results suggest that people who receive health information from the media are more likely to understand the negative health consequences of daily smoking and that this knowledge spurs them to reduce smoking frequency. We found that attention to health information from radio and newspaper sources also makes a person more likely to be an intermittent than former smoker. This finding indicates that media health messages may be misinterpreted as condoning intermittent smoking as a healthy alternative to daily smoking. If this is true, messages to heavy smokers to decrease smoking (32) could prevent intermittent smokers from complete cessation. Although these analyses are exploratory, they provide further evidence to support the links between communication inequalities and health outcomes as hypothesized by SIM (9) and indicate that further work in this field is warranted.

The cancer-related health beliefs investigated in this study did not differentiate intermittent from daily smokers. This finding suggests that these beliefs, mainly about the inevitability of cancer and the reversibility of damage from smoking, are not beliefs that would persuade daily smokers to change to intermittent smoking status. Other health beliefs, however, could reduce smoking frequency. The notion that environmental tobacco smoke is harmful to others may encourage people to smoke only when they are alone (33). Additionally, personal health issues such as heart disease or impotence may be more motivational than cancer in encouraging a person to reduce smoking frequency.

We found that cancer-related health beliefs can differentiate intermittent smokers and former smokers; those who believe that behavior influences cancer risk are more likely to stop smoking than to smoke intermittently. Those who believe that smoking damage cannot be reversed by healthy behaviors such as exercising and consuming vitamins are more likely to be former than intermittent smokers. To encourage cessation among intermittent smokers, a consistent message focusing on the permanent but avoidable damage caused by smoking could be an effective tool.

Several limitations in this article must be noted. These findings are cross-sectional and do not provide evidence of causality. The temporal ordering of media use, cancer-related health beliefs, and smoking status are unknown. A former smoker may have stopped smoking years ago, and his current high level of attention to health media may have resulted from unrelated recent illness. Because this data set contains no longitudinal data, it cannot provide information on smoking trends over time. Additionally, the data set cannot rule out the presence of selective attention by which, for example, daily smokers do not pay attention to health media to avoid messages incompatible with their smoking behavior. Finally, the binary variables used to measure attention to health media sources and cancer-related beliefs are exploratory measures that require further study to be validated.

This study has a number of strengths, including the use of a nationally representative data set of people who had ever smoked. This study is also unique in investigating the trends of intermittent smoking by using 2 measures of socioeconomic status and for investigating the association between health media use and smoking status.

This study has implications for public health practice. This work reinforces the notion that specific demographic groups may engage in long-term or frequent periods of intermittent smoking. Further work should be done to create effective campaigns that encourage cessation among intermittent smokers. Although our results should be interpreted cautiously, this study indicates that health media may increase the practice of intermittent smoking under some circumstances but could also be an effective tool to promote smoking cessation. Campaigns that promote health beliefs that portray damage caused by smoking as permanent but avoidable may increase cessation among intermittent smokers. Intermittent smoking could, however, be promoted as a temporary step toward complete smoking cessation among more disadvantaged groups that are less likely to stop smoking. Addressing these social disparities in health and health communication could improve the health of those most disadvantaged by communication inequalities and improve the health of the nation as a whole.

## Figures and Tables

**Table 1:** Socioeconomic, Demographic, Health Media Use, and Cancer Belief Variables Among People Who Had Ever Smoked, 2003 Health Information National Trends Survey

Variable	No. of Smokers (Weighted %)^a^			

	Total (N = 2,641)	Daily Smokers (n = 869)	Intermittent Smokers (n = 280)	Former Smokers (n = 1,492)
**Education (*P* < .001)**				
Less than high school graduate	352 (19.0)	133 (41.3)	33 ( 9.7)	186 (49.0)
High school graduate	859 (34.1)	351 (41.4)	96 (11.4)	412 (47.2)
Some college	778 (28.6)	258 (35.4)	90 (13.2)	430 (51.5)
College graduate	652 (18.3)	127 (19.4)	61 ( 8.9)	464 (71.7)
**Annual household income, $ (*P* < .001)**				
<25,000	854 (31.8)	346 (44.3)	90 (10.2)	418 (45.5)
25,000-34,999	411 (15.0)	152 (39.4)	49 (13.3)	210 (47.3)
35,000-49,999	427 (16.6)	143 (36.4)	51 (13.8)	233 (49.9)
50,000-74,999	432 (16.6)	122 (30.3)	42 (10.5)	268 (59.3)
≥75,000	517 (20.1)	106 (22.9)	48 ( 9.4)	363 (67.7)
**Marital status (*P* < .001)**				
Married or cohabitating	1,436 (63.7)	398 (29.6)	135 (9.7)	903 (60.8)
Not married or cohabitating	1,205 (36.3)	471 (46.2)	145 (13.8)	589 (40.0)
**Sex (*P* nonsignificant)**				
Male	1,228 (55.6)	373 (34.4)	125 ( 9.9)	730 (55.7)
Female	1,413 (44.4)	496 (37.1)	155 (12.7)	762 (50.2)
**Ethnicity (*P* < .01)**				
Non-Hispanic white	1,946 (75.6)	620 (35.1)	167 ( 9.3)	1,159 (55.6)
English-speaking Hispanic	172 ( 5.0)	57 (36.9)	34 (17.3)	81 (45.8)
Spanish-speaking Hispanic	103 ( 4.4)	20 (20.2)	33 (33.6)	50 (46.1)
Non-Hispanic black	258 ( 8.3)	104 (41.6)	32 (14.0)	122 (44.4)
Non-Hispanic otder	162 ( 6.8)	68 (43.6)	14 ( 8.6)	80 (47.8)
**Location of residence (*P* nonsignificant)**				
Metropolitan area of ≥1 million residents	1,286 (47.4)	387 (33.8)	142 (11.9)	757 (54.4)
Metropolitan area of <1 million residents	835 (32.7)	276 (34.5)	94 (12.1)	465 (53.5)
Nonmetroplitan area of ≥20,000 urban residents	192 ( 7.0)	71 (38.9)	21 ( 8.2)	100 (52.9)
Nonmetropolitan area of <20,000 urban residents	328 (12.9)	135 (43.6)	23 ( 7.7)	170 (48.7)
**Age, y (*P* < .001)**				
18-34	551 (25.1)	237 (47.1)	105 (19.8)	209 (33.1)
35-49	820 (32.3)	350 (45.6)	98 (11.6)	372 (42.8)
50-64	743 (25.2)	212 (27.2)	51 ( 6.2)	480 (66.6)
65-74	313 (10.9)	50 (15.6)	18 ( 6.1)	245 (78.3)
≥75	214 ( 6.5)	20 ( 7.7)	8 ( 3.0)	186 (89.4)
**History of cancer (*P* < .001)**				
No	2,262 (87.5)	761 (36.7)	252 (11.7)	1,249 (51.6)
Yes	379 (12.5)	108 (27.9)	28 ( 7.1)	243 (65.0)
**Family history of cancer (*P* nonsignificant)**				
No	908 (34.9)	282 (34.0)	114 (14.0)	512 (52.0)
Yes	1,733 (65.1)	587 (36.5)	166 (9.6)	980 (53.9)
**Information seeking (*P* nonsignificant)**				
Does not seek	1,399 (56.0)	480 (37.3)	158 (12.1)	761 (50.5)
Seeks	1,242 (44.0)	389 (33.5)	122 (9.9)	731 (56.7)
**Attention paid to television (*P* nonsignificant)**				
A lot	871 (30.7)	264 (32.5)	103 (12.7)	504 (54.8)
Not a lot	1,770 (69.4)	605 (37.0)	177 (10.5)	988 (52.6)
**Attention paid to radio (*P* < .05)**				
A lot	408 (14.2)	118 (29.7)	55 (16.9)	235 (53.4)
Not a lot	2,233 (85.9)	751 (36.6)	225 (10.2)	1,257 (53.2)
**Attention paid to newspapers (*P* nonsignificant)**				
A lot	634 (22.7)	182 (31.2)	69 (12.3)	383 (56.5)
Not a lot	2,007 (77.3)	687 (36.9)	211 (10.8)	1109 (52.3)
**Attention paid to magazines (*P* nonsignificant)**				
A lot	626 (21.1)	181 (30.1)	70 (11.9)	375 (58.0)
Not a lot	2,015 (78.9)	688 (37.1)	210 (10.9)	1,117 (52.0)
**Attention paid to the Internet (*P* nonsignificant)**				
A lot	332 (12.0)	115 (39.9)	36 ( 9.9)	181 (50.3)
Not a lot	2,309 (88.0)	754 (35.0)	244 (11.3)	1,311 (53.7)
**Exercise can undo most effects of smoking (*P* < .001)**				
Disagrees	1,398 (50.8)	394 (29.6)	128 (10.0)	876 (60.4)
Does not disagree	1,243 (49.2)	475 (41.9)	152 (12.3)	616 (45.8)
**Vitamins can undo most effects of smoking (*P* < .05)**				
Disagrees	1,744 (64.9)	516 (32.2)	172 (10.7)	1,056 (57.1)
Does not disagree	897 (35.1)	353 (42.1)	108 (11.9)	436 (46.1)
**No risk of cancer if someone only smokes a few years (*P* nonsignificant)**				
Disagrees	2,127 (80.4)	705 (36.1)	214 (11.0)	1,208 (52.9)
Does not disagree	514 (19.6)	164 (33.6)	66 (11.8)	284 (54.6)
**Lung cancer depends more on genes (*P* nonsignificant)**				
Disagrees	1,574 (59.6)	488 (34.7)	153 (10.8)	933 (54.5)
Does not disagree	1,067 (40.4)	381 (37.0)	127 (11.6)	559 (51.4)
**Almost everything causes cancer (*P* < .001)**				
Disagrees	1,095 (40.0)	306 (30.1)	96 ( 8.8)	693 (61.1)
Does not disagree	1,546 (60.0)	563 (39.3)	184 (12.7)	799 (48.0)
**Not much people can do to lower risk of cancer (*P* nonsignificant)**				
Disagrees	1,699 (63.0)	535 (33.6)	185 (11.3)	979 (55.0)
Does not disagree	942 (37.0)	334 (39.0)	95 (10.8)	513 (50.2)

a Percentages add to 100 vertically within each variable for the "total" column and horizontally for the 3 columns of smoking status. *P* values are for χ^2^ tests for cross-tabulation between each variable and the 3 categories of smoking status.

**Table 2:** Multivariate Analysis of Socioeconomic and Demographic Variables by Smoking Status, 2003 Health Information National Trends Survey

Variable	OR (95% CI)^a^	

	Intermittent vs Former Smoker	Intermittent vs Daily Smoker
**Education**		
Less than high school graduate	1 [Reference]	1 [Reference]
High school graduate	1.20 (0.56-2.55)	1.53 (0.69-3.41)
Some college	1.26 (0.63-2.51)	1.92 (0.85-4.32)
College graduate	0.82 (0.43-1.56)	2.30 (1.07-4.93)
**Annual household income, $**		
<25,000	1 [Reference]	1 [Reference]
25,000-34,999	1.28 (0.70-2.31)	1.77 (0.85-3.68)
35,000-49,999	1.38 (0.72-2.66)	2.27 (1.14-4.51)
50,000-74,999	0.84 (0.48-1.49)	2.08 (1.12-3.85)
≥75,000	0.84 (0.44-1.63)	2.51 (1.23-5.13)
**Marital status**		
Married or cohabitating	1 [Reference]	1 [Reference]
Not married or cohabitating	1.90 (1.27-2.83)	1.17 (0.76-1.80)
**Sex**		
Male	1 [Reference]	1 [Reference]
Female	1.56 (1.12-2.18)	1.67 (1.10-2.52)
**Ethnicity**		
Non-Hispanic white	1 [Reference]	1 [Reference]
English-speaking Hispanic	1.39 (0.76-2.55)	1.73 (0.82-3.62)
Spanish-speaking Hispanic	2.91 (1.39-6.10)	12.74 (5.08-31.92)
Non-Hispanic black	1.48 (0.81-2.69)	1.57 (0.82-3.00)
Non-Hispanic other	1.02 (0.47-2.23)	0.88 (0.44-1.73)
**Location of residence**		
Metropolitan area of ≥1 million residents	1 [Reference]	1 [Reference]
Metropolitan area of <1 million residents	1.25 (0.82-1.92)	1.38 (0.89-2.15)
Nonmetroplitan area of ≥20,000 urban residents	0.96 (0.45-2.01)	0.97 (0.47-2.00)
Nonmetropolitan area of <20,000 urban residents	1.05 (0.53-2.08)	0.81 (0.37-1.74)
**Age, y**		
18-34	1 [Reference]	1 [Reference]
35-49	0.56 (0.34-0.93)	0.63 (0.38-1.05)
50-64	0.20 (0.11-0.37)	0.56 (0.30-1.04)
65-74	0.15 (0.07-0.30)	1.15 (0.46-2.85)
≥75	0.05 (0.02-0.14)	1.35 (0.42-4.36)
**History of cancer**		
No	1 [Reference]	1 [Reference]
Yes	1.04 (0.57-1.88)	0.87 (0.46-1.65)
**Family history of cancer**		
No	1 [Reference]	1 [Reference]
Yes	0.81 (0.56-1.17)	0.76 (0.49-1.18)
**Information seeking**		
Does not seek	1 [Reference]	1 [Reference]
Seeks	0.75 (0.50-1.13)	0.92 (0.59-1.42)

Abbreviations: OR, odds ratio; CI, confidence interval.

a ORs indicate the relative likelihood of being an intermittent smoker rather than a former smoker or a daily smoker for a given level of the exposure variable compared with the reference level of the exposure variable. For example, a Spanish-speaking Hispanic person has nearly 3 times the odds of being an intermittent rather than a former smoker compared with a non-Hispanic white person.

**Table 3:** Multivariate Analysis of Attention Paid to Various Types of Health Media by Smoking Status, 2003 Health Information National Trends Survey

Health Media Type^a^	OR (95% CI)^b^	

	Intermittent vs Former Smoker	Intermittent vs Daily Smoker
**Television**		
Not a lot of attention	1 [Reference]	1 [Reference]
A lot of attention	1.20 (0.85-1.70)	1.36 (0.95-1.95)
**Radio**		
Not a lot of attention	1 [Reference]	1 [Reference]
A lot of attention	1.68 (1.01-2.80)	2.12 (1.27-3.56)
**Newspapers**		
Not a lot of attention	1 [Reference]	1 [Reference]
A lot of attention	1.48 (1.02-2.14)	1.47 (0.94-2.30)
**Magazines**		
Not a lot of attention	1 [Reference]	1 [Reference]
A lot of attention	0.92 (0.61-1.41)	1.34 (0.94-1.92)
**Internet**		
Not a lot of attention	1 [Reference]	1 [Reference]
A lot of attention	0.78 (0.48-1.26)	0.77 (0.43-1.37)

Abbreviations: OR, odds ratio; CI, confidence interval.

a Each health media type was analyzed in a different logistic regression model, and all models were adjusted for education, annual household income, marital status, sex, race/ethnicity, location of residence, age, history of cancer, and family history of cancer.

b ORs indicate the relative likelihood of being an intermittent smoker rather than a former smoker or a daily smoker for a given level of the exposure variable compared with the reference level of the exposure variable. For example, a person who pays a lot of attention to health information on the radio has more than twice the odds of being an intermittent rather than a daily smoker compared with a person who does not pay a lot of attention.

**Table 4:** Multivariate Analysis of Cancer-Related Health Beliefs by Smoking Status, 2003 Health Information National Trends Survey

Cancer-Related Health Belief^a^	OR (95% CI)^b^	

	Intermittent vs Former Smoker	Intermittent vs Daily Smoker
**Exercise can undo most effects of smoking**		
Does not disagree	1 [Reference]	1 [Reference]
Disagrees	0.48 (0.32-0.72)	1.24 (0.86-1.80)
**Vitamins can undo most effects of smoking**		
Does not disagree	1 [Reference]	1 [Reference]
Disagrees	0.69 (0.49-0.98)	1.19 (0.80-1.78)
**No risk of cancer if someone only smokes a few years**		
Does not disagree	1 [Reference]	1 [Reference]
Disagrees	0.85 (0.57-1.26)	0.88 (0.50-1.55)
**Lung cancer depends more on genes**		
Does not disagree	1 [Reference]	1 [Reference]
Disagrees	0.72 (0.52-0.99)	0.93 (0.65-1.33)
**Almost everything causes cancer**		
Does not disagree	1 [Reference]	1 [Reference]
Disagrees	0.66 (0.47-0.93)	0.80 (0.56-1.15)
**Not much people can do to lower risk of cancer**		
Does not disagree	1 [Reference]	1 [Reference]
Disagrees	1.01 (0.72-1.42)	1.14 (0.80-1.63)

Abbreviations: OR, odds ratio; CI, confidence interval.

a Each cancer-related health belief was analyzed in a different logistic regression model, and all models were adjusted for education, annual household income, marital status, sex, race/ethnicity, location of residence, age, history of cancer, and family history of cancer.

b ORs indicate the relative likelihood of being an intermittent smoker rather than a former smoker or a daily smoker for a given level of the exposure variable compared with the reference level of the exposure variable. For example, a person who disagrees with the assertion that exercise can undo the damage from smoking has less than half the odds of being an intermittent rather than a former smoker compared with a person who does not disagree with that assertion.
